# LC-MS/MS Determination and Pharmacokinetic Study of Pedunculoside in Rat Plasma after Oral Administration of Pedunculoside and *Ilex rotunda* Extract

**DOI:** 10.3390/molecules20059084

**Published:** 2015-05-19

**Authors:** Waiou Zhao, Li Pang, Dahai Xu, Nan Zhang

**Affiliations:** 1 Cardiovascular Medicine, the First Hospital of Jilin University, Changchun 130021, China; E-Mail: zhao241241241@sina.cn; 2 Emergency Department, the First Hospital of Jilin University, Changchun 130021, China; E-Mails: qw_ywyq4479@126.com (L.P.); sah211@sina.cn (D.X.)

**Keywords:** LC-MS/MS, pedunculoside, pharmacokinetics, *Ilex rotunda*

## Abstract

*Ilex rotunda* is widely used to treat many disorders as a traditional Chinese medicine (TCM) containing 4%–5% pedunculoside (PDC). A rapid, selective, and sensitive liquid chromatography-tandem mass spectrometry method (LC-MS/MS) was developed and validated to determine PDC in rat plasma by using 3β,19α-dihydroxyurs-12-en-28-oic acid 28-β-D-glucopyranosyl ester (DEOG) as an internal standard. The analytes were extracted by protein precipitation and eluted on a C_18_ chromatography column using a mobile phase of methanol–H_2_O (70:30, *v/v*) delivered at a flow rate of 0.6 mL/min. Detection was performed using positive ion electrospray ionization in multiple reaction monitoring modes. The assay was linear over the concentration range of 0.60 ng/mL to 200 ng/mL, with a quantification limit of 0.60 ng/mL. Intra-day and inter-day precisions (%RSD) ranged from 2.12 to 9.51 for PDC, whereas the accuracy was within −7.83%~9.40%. The validated method was successfully applied to the pharmacokinetic study of PDC in rat plasma after oral administration of pure PDC and *Ilex rotunda* extract (IRE). Pharmacokinetic parameters of PDC in IRE, such as *C_max_*, *AUC_0–t_*, *AUC_0–∞_*, *t_1/2z_*_,_ and *CL*_z_/*F*, statistically differed from those of the pure monomer (*p* < 0.01). However, *T_max_* and *MRT* showed no significant differences between the two groups. Results suggested that other coexisting components in IRE may decrease the absorption of PDC. Compound-compound interactions between PDC and other herbal extract components can alter the pharmacokinetic behavior of PDC. The study will be helpful in providing references for understanding the action mechanism and clinical application of *Ilex rotunda*.

## 1. Introduction

Traditional Chinese medicine (TCM) uses natural therapeutic agents in accordance with the TCM science theory, which has been applied by TCM practitioners for centuries. To date, with improving methods and advanced technology, increasing attention has been given to TCM. The genus *Ilex* (Aquifoliaceae), which includes approximately 500 to 600 species of trees and shrubs, are widely distributed in tropical and subtropical regions worldwide [1]. For example, the barks of *Ilex rotunda*, which is called “Jiu Bi Ying” in China, are officially recorded in the Chinese Pharmacopoeia and used to treat common colds, tonsillitis, eczema, and stomach and intestinal ulcers [2]. Previous studies have shown that the *Ilex rotunda* extract (IRE) has garnered considerable attention for its anti-inflammatory [3], antihypertensive [4], antibacterial [5], and antioxidant activities [6], as well as cardiovascular protection effects [7,8].

**Figure 1:**
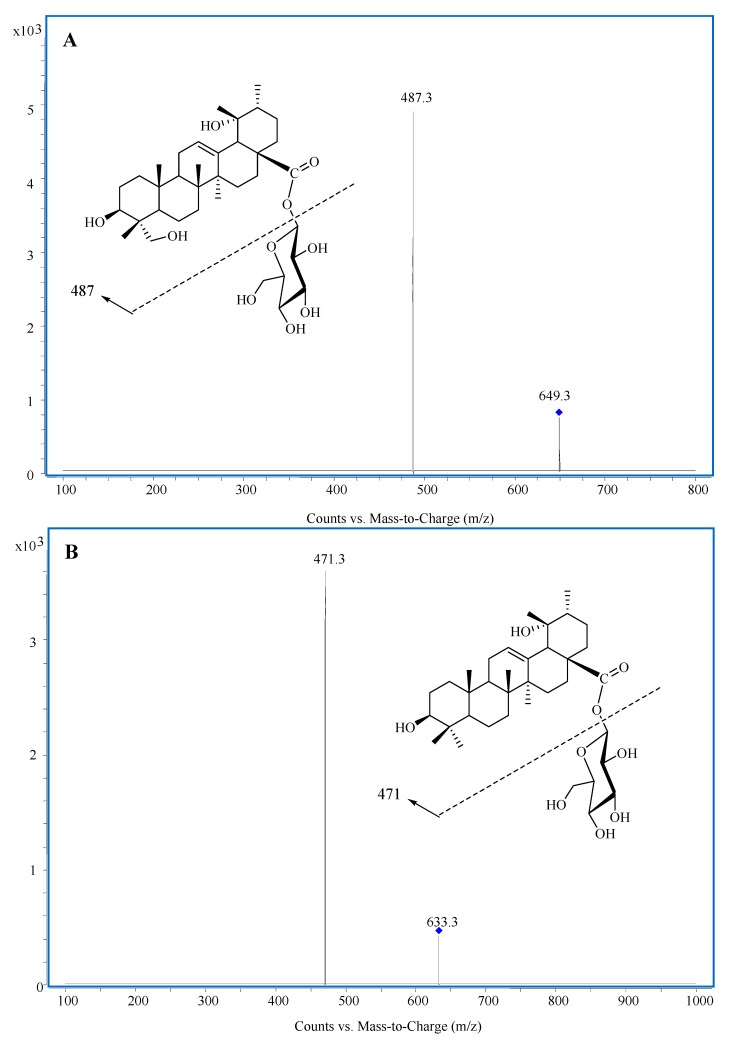
Representative MS/MS spectra and structures: (**A**) PDC; (**B**) IS.

Pharmacokinetic studies on active ingredients of herbal extracts in TCM are important to illustrate their mechanisms of action. Previous phytochemical studies on the barks and leaves of *Ilex rotunda* have resulted in the isolation of triterpenes [9], triterpene saponins [10,11], phenylpropanoids [12], and hemiterpene glycosides [13]. Triterpene saponins are commonly recognized as major constituents of the *Ilex* species and have been proven to be major active constituents closely related to the pharmacological cardiovascular protection activities [14,15,16,17]. Pedunculoside (PDC, Figure 1A), a triterpene saponin, is a representative and abundant component of *Ilex rotunda* [18,19]. This compound showed significant hypolipidemic activity on hyperlipidemic albino rats [20]. PDC has been used as a phytochemical marker for the quality control of *Ilex rotunda* in the Chinese Pharmacopoeia, and the content of PDC in medicines should not be less than 4.5% [2].

Given the complexity of chemicals in herbal medicines, the pharmacokinetic profile of one major active ingredient will possibly be altered by coexisting components in the herbal extract [21,22]. Several methods for determining the presence of PDC in the genus *Ilex* and other herbal materials have used high-speed counter-current chromatography [23], high-performance liquid chromatography coupled with ultraviolet detection (HPLC-UV) [24,25], HPLC coupled with evaporative light scattering detection [26,27], and ultra-performance liquid chromatography/quadrupole time-of-flight mass spectrometry (UPLC/Q-TOF-MS) [28]. However, analysis of PDC *in vivo* has yet to be reported. Furthermore, the pharmacokinetic behavior of PDC has not been investigated after oral administration of crude IRE either. Thus, whether other ingredients can affect the pharmacokinetic behavior of PDC should be explored. In the present study, a selective and sensitive liquid chromatography-tandem mass spectrometry (LC-MS/MS) assay for analyzing PDC in rat plasma was developed for the first time. We compared the pharmacokinetics of PDC in rats after oral administration of PDC alone and with IRE. This study will be useful to explain and predict various events related to the efficacy and toxicity of drugs, as well as provide a firm basis for the design of dosage regimens in pharmacodynamic experiments and preclinical safety evaluation.

## 2. Results and Discussion

### 2.1. Optimization of LC/MS/MS Conditions

PDC and other components exist in the IRE, however, the co-existing components do not influence the accurate detection of PDC owing to the highly selective and accurate MRM scan of the mass spectrometer. Method validation was done for PDC alone in our experiment, without considering the other co-existing compounds in the IRE.

To optimize the electrospray ionization (ESI) conditions for PDC and internal standard (IS), quadrupole full scans were performed in the positive or negative detection mode by infusing approximately 1000 ng/mL of analyte in acetonitrile. After the ESI full scan tested, the results showed that ESI in the negative ion mode provided a higher sensitivity. PDC and IS gave predominantly a singly charged deprotonated precursor [M−H]^−^ at *m*/*z* 649.3 and 633.3 in Q1 full scan mode, respectively. The major fragment ions appreared in the product spectrum at *m*/*z* 487.3 for PDC when the collision energy was 15 eV (Figure 1). Thus the *m*/*z* 487.3 fragment was used for the quantification of PDC. Under similar conditions, the optimum multiple reaction monitoring (MRM) transition of IS was chosen as *m*/*z* 633.3→471.3 when the collision energy was 12 eV.

Analysis was performed on several different HPLC columns, including a ZORBAX Eclipse C_18_ column (50 mm × 4.6 mm, 5 µm), Hypersil GOLD C_18_ column (50 mm × 4.6 mm, 3 µm) and Agela Venusil MP C_18_ column (50 mm × 4.6 mm, 3 µm). The Hypersil GOLD C18 column (50 mm × 4.6 mm, 3 µm) demonstrated symmetrical peak shapes and better separation for the analyte and IS compared with the other chromatographic columns. Several variables, including the percentage of acetonitrile/water, methanol/water and acidic modifier in the mobile phase were selected for the optimization procedure. The presence of methanol in the mobile phase significantly improved the response and resolution of the analytes, and the final optimized volume ratio of methanol-water was 70:30 (*v/v*).

### 2.2. Selection of IS

It is necessary to use an appropriate IS to get high accuracy when a mass spectrometer equipped with HPLC system is used as the detector. To find a compound that could ideally mirror the analyte and serve as an appropriate IS, we screened several compounds and 3β,19α-dihydroxyurs-12-en-28-oic acid 28-β-D-glucopyranosyl ester (DEOG, Figure 1) was finally chosen for quantification as the IS due to its similarity with the analyte in structure, mass spectrographic and chromatographic behaviors.

### 2.3. Method Validation

#### 2.3.1. Specificity and Matrix Effect

**Figure 2:**
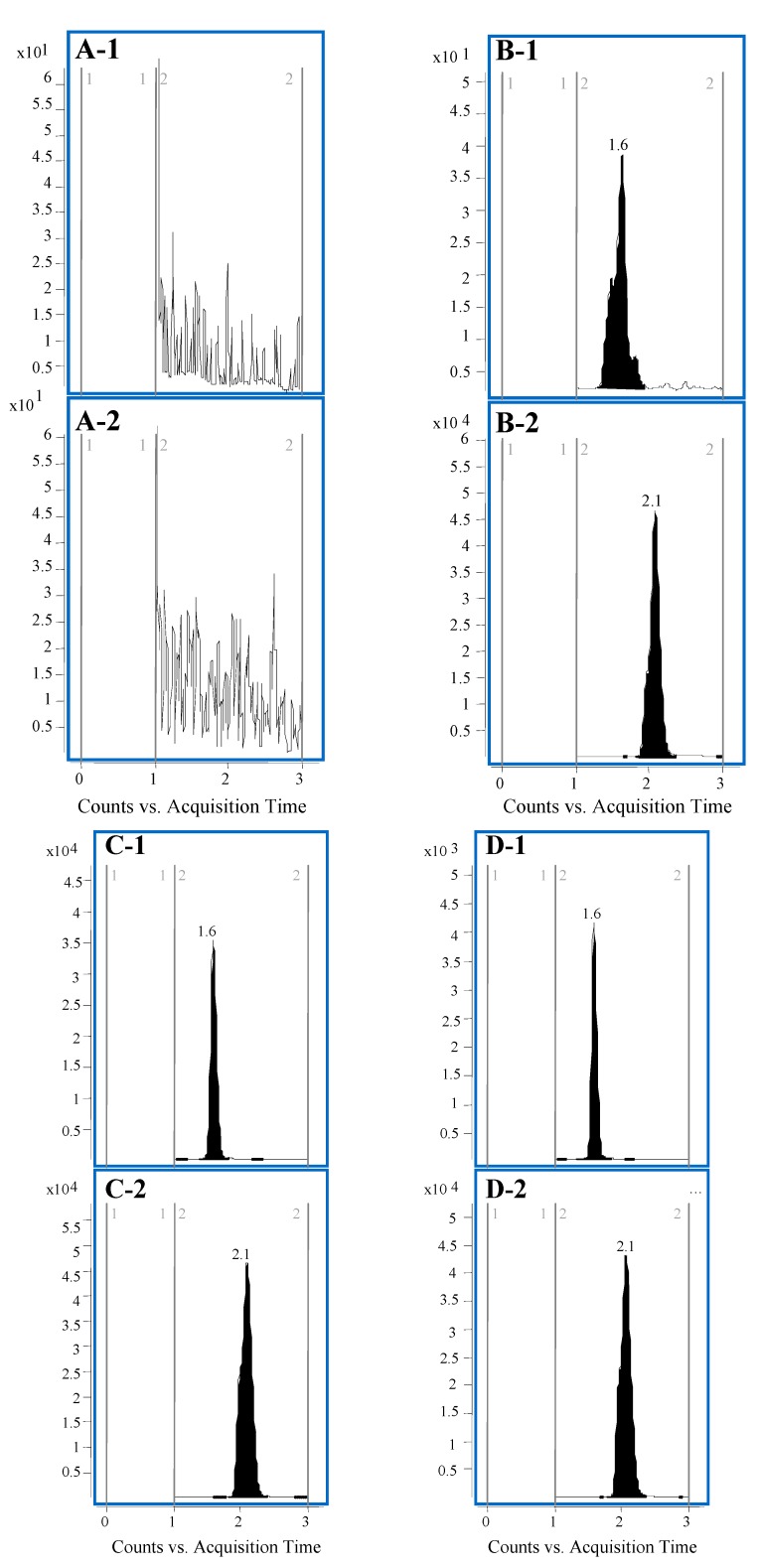
Typical LC-MS/MS chromatograms: (**A**) blank plasma; (**B**) blank plasma spiked with PDC (2.0 ng/mL) and IS (200 ng/mL); (**C**) rat plasma after oral administration of pure PDC; (**D**) rat plasma after oral administration of IRE. 1: PDC; 2: IS.

As shown in Figure 2, no significant endogenous interference is observed at the retention times of PDC and IS, which were 1.6 and 2.1 min, respectively. The matrix effect values were 94.32% ± 5.24%, 95.63% ± 3.90%, and 99.10% ± 5.04% for PDC at the four QC concentration levels. The average matrix effect value of IS was 94.68% ± 2.57%. No significant matrix effect was observed for PDC and IS, indicating that no co-eluting substance would influence the ionization of the analytes.

#### 2.3.2. Precision and Accuracy

Intra-day and inter-day precision and accuracy were established from validation runs performed at four QC levels (Table 1). The intra-day precision (relative standard deviation, RSD) ranged from 4.87% to 9.51% for PDC while the accuracy (relative error, RE) was within −7.83%~8.67%. Similarly, for the inter-day experiments, the precision varied from 2.12% to 6.41% for PDC while the accuracy was within −1.64%~9.40%.

**Table 1:** Intra- and inter-day accuracy and precision for determination of PDC in rat plasma.

Nominal Conc. (ng/mL)	Intra-Day (*n* = 6)			Inter-Day (*n* = 18)		
	Determined Conc. (Mean ± SD, ng/mL)	RE (%)	RSD (%)	Determined Conc. (Mean ± SD, ng/mL)	RE (%)	RSD (%)
0.60 (LLOQ)	0.652 ± 0.062	8.67	9.51	0.614 ± 0.013	2.33	2.12
1.50 (QC-low)	1.38 ± 0.10	−7.83	6.87	1.61 ± 0.04	7.08	2.62
15.0 (QC-medium)	15.16 ± 1.40	1.08	9.06	16.41 ± 1.05	9.40	6.41
180 (QC-high)	171.53 ± 8.36	−4.71	4.87	177.04 ± 10.51	−1.64	5.93

#### 2.3.3. Calibration Curve and Sensitivity

All the calibration curves showed good linearity over the concentration range of 0.60–200 ng/mL in rat plasma. A typical equation of the calibration curves was y = 8.6689 × 10^−4^ x + 2.3973 × 10^−4^ (r^2^ = 0.9970), where y was the peak area ratio of PDC to IS and x was the concentration of PDC. The lower limit of quantitation (LLOQ) for PDC in rat plasma was 0.60 ng/mL, which was sufficient for the pharmacokinetic study.

#### 2.3.4. Recovery

The extraction recovery of PDC in rat plasma at four levels of QC samples ranged from 92.60% to 95.11% (Table 2), the mean recovery was 93.21% with the RSD of 4.61% and 7.29%, and the mean extraction efficiency of IS was 91.82%, which could meet the requirements of analysis.

**Table 2:** The recovery for determination of PDC in rat plasma.

Analyte	Nominal Conc. (ng/mL)	Recovery (%, *n* = 6)	
		Mean	RSD (%)
PDC	0.60 (LLOQ)	92.60	7.29
	1.50 (QC-low)	94.34	4.61
	15.0 (QC-medium)	94.66	5.48
	180 (QC-high)	95.11	5.31
IS	200	91.82	3.08

#### 2.3.5. Stability

The stability of PDC was evaluated by exposing it to different conditions (time and temperature) at four QC concentration levels (LLOQ, QC-low, QC-medium, and QC-high) in triplicate. The results were compared with those obtained with freshly prepared QC samples. All the stability test results are listed in Table 3. It could be seen from Table 3 that the results are well within the acceptance limits.

**Table 3:** The stability results for determination of PDC in rat plasma (*n* = 6).

Storage Conditions	PDC			
	Nominal Conc. (ng/mL)	Determined Conc. (Mean ± SD, ng/mL)	RE (%)	RSD (%)
Short-term (25 °C for 4 h)	0.60 (LLOQ)	0.673 ± 0.014	12.24	2.04
	1.50 (QC-low)	1.44 ± 0.09	−3.77	6.10
	15.0 (QC-medium)	14.88 ± 1.03	−0.77	6.94
	180 (QC-high)	192.71 ± 11.42	7.06	5.93
Long-term stability (−20 °C for 2 months)	0.60(LLOQ)	0.573 ± 0.008	−4.56	1.40
	1.50 (QC-low)	1.52 ± 0.16	1.11	10.27
	15.0 (QC-medium)	15.96 ± 0.68	6.39	4.29
	180 (QC-high)	160.70 ± 7.58	−10.72	4.72
Three freeze/thaw cycles (room temperature to −20 °C)	0.60 (LLOQ)	0.570 ± 0.016	−5.00	2.73
	1.50 (QC-low)	1.38 ± 0.162	−7.82	11.72
	15.0 (QC-medium)	16.32 ± 1.37	8.81	8.39
	180 (QC-high)	162.85 ± 15.57	−9.53	9.56
Post-preparation stability (4 °C for 12 h)	0.60 (LLOQ)	0.596 ± 0.011	−0.60	1.91
	1.50 (QC-low)	1.65 ± 0.10	10.04	6.32
	15.0 (QC-medium)	13.81 ± 0.63	−7.91	4.54
	180 (QC-high)	183.17 ± 2.59	1.76	1.41

### 2.4. Pharmacokinetic Study

The analytical procedures described in Section 3.3 were performed to quantify PDC in rat plasma samples obtained from 24 male Sprague-Dawley rats. Out of these rats, 12 were orally administered with pure PDC, whereas 12 were orally administered with IRE.

**Figure 3:**
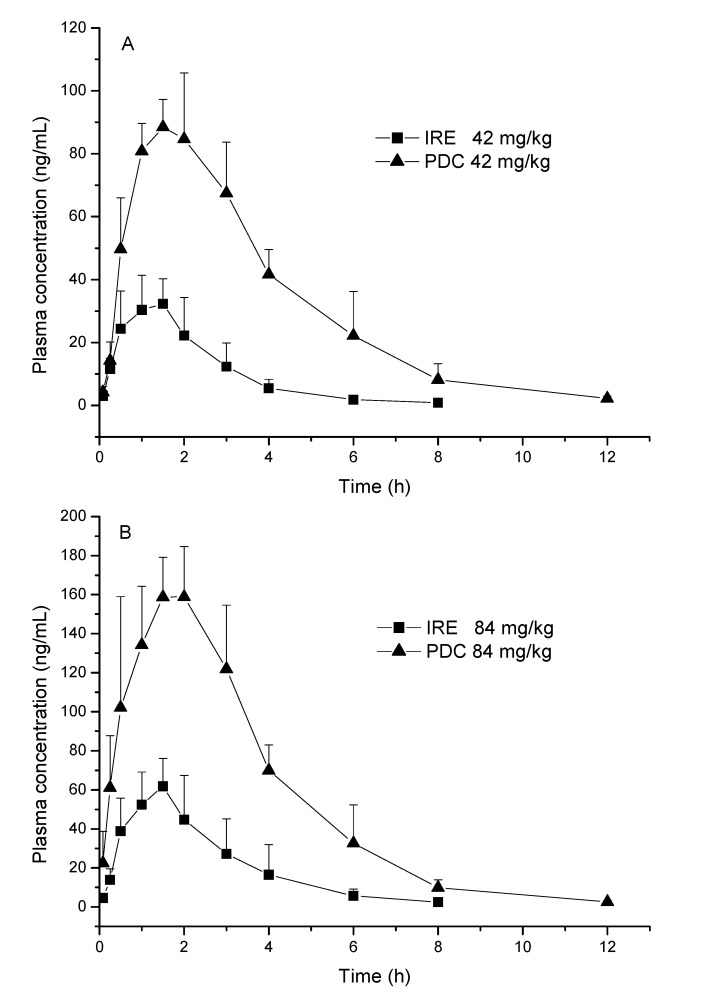
Mean plasma concentration-time profiles of PDC in rat plasma after oral administration of pure PDC or the IRE (dosage of IRE was equivalent to corresponding PDC dosage) (*n* = 6). (**A**) plot for 42 mg/kg PDC and IRE; (**B**) plot for 84 mg/kg PDC and IRE.

Establishing an intravenous dose group might provide the bioavailability of PDC, however, the present experiment aimed to compare the pharmacokinetic differences of PDC between monomer and IRE groups after oral administration. A fully validated LC-MS/MS method was developed and successfully applied to the pharmacokinetic behavior of PDC in rats after oral administration of the pure PDC and IRE, which were given at the same equivalent doses (42 and 84 mg/kg PDC). The mean plasma concentration-time profiles of PDC and the herbal extract after oral administration at different doses are illustrated in Figure 3, and the estimated pharmacokinetic parameters are presented in Table 4.

As shown in Table 4, after the oral administration of 42 and 84 mg/kg, the PDC monomer was absorbed and reached a maximum concentration (*C_max_*) of (95.21 ± 7.93) and (174.39 ± 12.93) ng/mL, respectively. The *C_max_* of PDC from IRE at the same doses were (37.28 ± 5.69) and (69.07 ± 7.07) ng/mL, respectively.

The area under the plasma concentration *versus* time curve from zero to time *t* (*AUC_0–t_*) of PDC was (373.54 ± 82.94) µg·h/L at a dose of 42 mg/kg, which was lower than its *AUC_0–t_* (660.89 ± 149.20 µg·h/L) following a dose of 84 mg/kg. The *AUC_0–t_* of PDC in IRE was (82.78 ± 24.02) and (172.75 ± 65.65) µg·h/L at doses of 42 and 84 mg/kg, respectively. These results indicate that the values of *C_max_* and *AUC_0–t_* of PDC from IRE were lower than those from the pure PDC at the same dosage. However, for clearance (*CL*_z_/*F*) and apparent volume of distribution (*V*_z_/*F*), the parameters from IRE were higher than those of pure PDC at the same dosage.

**Table 4:** Pharmacokinetic parameters of PDC in rat plasma after oral administration of pure PDC or the IRE (42 and 84 mg/kg) (*n* = 6).

Parameters	42 mg/kg		84 mg/kg	
	Pure PDC	IRE ^a^	Pure PDC	IRE ^a^
*T_max_* (h)	1.60 ± 0.42	1.30 ± 0.27	1.80 ± 0.91	1.43 ± 0.22
*C_max_* (ng/mL)	95.21 ± 7.93	37.28 ± 5.69 **	174.39 ± 12.93	69.07 ± 7.07 **
*AUC_0–t_* (µg·h/L)	373.54 ± 82.94	82.78 ± 24.02 **	660.89 ± 149.20	172.75 ± 65.65 **
*AUC_0–∞_* (µg·h/L)	378.92 ± 84.79	85.10 ± 25.50 **	665.28 ± 151.12	176.72 ± 66.48 **
*MRT_0–t_*	3.17 ± 0.45	2.18 ± 0.36	3.08 ± 0.42	2.20 ± 0.53
*MRT_0–∞_*	3.33 ± 0.48	2.55 ± 0.40	3.15 ± 0.44	2.36 ± 0.56
*t_1/2z_* (h)	1.73 ± 0.11	1.07 ± 0.15 **	1.50 ± 0.08	1.11 ± 0.21 **
*CL*_z_/*F* (L/kg/h)	115.93 ± 28.60	534.75 ± 175.06 **	126.83 ± 19.81	534.92 ± 204.14 **
*V*_z_/*F* (L/h)	287.21 ± 63.55	831.22 ± 316.70 **	273.31 ± 45.57	930.56 ± 382.75 **

^a^ Dosage of IRE was equivalent to corresponding PDC dosage; Values are mean ± SD; ** *p* < 0.01 compared with the level of PDC at the same dose.

Pharmacokinetic parameters of PDC in IRE, such as *C_max_*, *AUC_0_*_–*t*_, the area under the plasma concentration *versus* time curve from zero to infinity (*AUC_0–∞_*), elimination half-life (*t_1/2z_*), and *CL*_z_/*F*, differed statistically from those of pure PDC (*p* < 0.01). However, the time of peak concentration (*T_max_*) and the mean residence time (*MRT*) showed no significant differences between the two groups. Notably, the significant differences in C*_max_*, *AUC_0_*_–*t*_, and *AUC_0_*_–*∞*_ suggested that other complex ingredients may exist in IRE, which can reduce the plasma contents of PDC by inhibiting absorption. The mechanism that accounts for the difference in pharmacokinetic behavior between pure PDC and IRE is unclear. However, compound-compound interactions in the IRE may present a main possible explanation. Many research have demonstrated the alteration in the pharmacokinetics of the target compound upon the use of pure monomer or the herbal extract [21,22,29]. Hence, a comparative pharmacokinetic study would provide more useful information for further understanding the mechanism of action and clinical application of the herbs. Moreover, the *t_1/2z_* of PDC in the extract group was shorter than that of the monomer group (*p* < 0.01), indicating that PDC in the extract was more easily eliminated. Therefore, the promotion of elimination may mainly be attributed to the existence of other compounds in the extract.

The *CL_z_*/*F* increases significantly with the extract, which may originate from *CL_z_* and *F*. If the clearance (*CL_z_*) increases, the *MRT* and *t_1/2z_* will decrease accordingly. Our data indicated that the parameters of *MRT* and *t_1/2z_* of the extract group were definitely lower than those of the PDC group, suggesting that *CL_z_* should be higher in the extract group. Furthermore, *F* is a parameter involved in the rate and extent of compound absorption. The *T_max_* values of pure PDC and IRE are similar, but the *C_max_* or *AUC* of the extract is considerably lower than that of pure PDC. The absorption of PDC in the extract is also slower than that of pure PDC. Thus, the *F* of PDC reduced when administered as an extract and significantly increased the *CL_z_*/*F* in extract form.

The current study is the first to investigate in rats the pharmacokinetic behavior of PDC, which is the indicative compound of IRE. The results will facilitate future studies on the pharmacokinetic study of possible compound-compound interactions in IRE and benefit more applications of this herbal medicine. Further investigation on the interaction mechanism of this substance is also necessary.

## 3. Experimental Section

### 3.1. Standards and Chemicals

The reference standards of PDC (98.5%) were purchased from the National Institutes for Food and Drug Control (Beijing, China), and DEOG (99.1%) used as the IS in this study, was obtained from Chengdu Chroma-biotechnology Co., Ltd (Chengdu, China). Water used for the LC/MS/MS analysis was prepared by using Milli-Q water purification system (Millipore, Bedford, MA, USA). HPLC grade methanol was purchased from Fisher Scientific (Pittsburgh, PA, USA). The blank rat plasma sample was prepared in the Laboratory Animal Center of Jilin University (Changchun, China).

### 3.2. Animals

Male Sprague-Dawley rats (220–250 g) were purchased from Laboratory Animal Center of Jilin University (Changchun, China) used to study the pharmacokinetics of PDC. All 24 rats were kept for 7 days in a controlled environment at 22 ± 2 °C and 45% ± 10% relative humidity with free access to water.

### 3.3. Instrumentation and Conditions

LC-MS/MS was performed using an Agilent 1200 series HPLC equipped with a ThermoFisher Scientific Hypersil GOLD C_18_ column (50 mm × 4.6 mm, i.d., 3 µm) maintained at 30 °C and an Agilent 6460 Triple Quadrupole mass spectrometer equipped with an ESI source (Agilent Technologies, Santa Clara, CA, USA). The mass spectrometer was operated in negative ionization mode. All data were acquired and processed using Agilent 6460 Quantitative Analysis processing software.

The mobile phase was methanol–H_2_O (70:30, *v/v*) delivered at a flow rate of 0.6 mL/min. Detection was by MRM of the precursor-to-product ion transitions at *m*/*z* 649.3–487.3 for PDC and *m*/*z* 633.3–471.3 for IS. Optimized MS parameters were as follows: drying gas temperature 300 °C; dry gas flow rate 10 L/min; nebulizer pressure 35 psi; dwell time per transition 200 ms; EMV (–)200 V; fragmentor voltages 145 V (PDC) and 135 V (IS); collision energies 15 eV (PDC) and 12 eV (IS).

### 3.4. Preparation of Ilex rotunda Extract Powder

For preparation of the extract, the dried powder of *Ilex rotunda* barks (50 g) was extracted three times under reflux with 600 mL ethanol-water (50:50, *v/v*) for 1.5 h each time, and then filtered [17,18]. The solution was then concentrated to 50 mL using a rotary evaporator and stored at −20 °C overnight for freezing. The frozen decoctions were freeze dried by a lyophilizer to obtain the dry powder containing the total glycosides (8.55 g). The content of PDC in the total glycoside powder was quantified using the same chromatography conditions as described in Section 3.3. The results showed that the content of PDC in the extract powder was 26.3%, which was close to the previous reported values [18,19].

### 3.5. Preparation of Calibration Standards and Quality Control Samples

A stock solution of PDC (1.0 mg/mL) was prepared in methanol and serially diluted with methanol to give standard solutions with concentrations of 12.0, 40.0, 120, 400, 1200 and 4000 ng/mL. In a similar manner, an IS working solution (200 ng/mL) was prepared by diluting a stock solution of DEOG (1.0 mg/mL). All solutions were kept at −20 °C until use. Calibration standards (0.60, 2.00, 6.00, 20.0, 60.0 and 200.0 ng/mL) were prepared by spiking 10 μL of each standard solution with an aliquot of 190 μL blank rat plasma. Quality control (QC) samples were prepared in the same way at four concentrations of 0.60 ng/mL (LLOQ), 1.50 ng/mL (QC-low), 15.0 ng/mL (QC-medium), and 180 ng/mL (QC-high). The calibration standards and the QC samples were used in the assay validation and the pharmacokinetic study.

### 3.6. Biosample Preparation

A simple and rapid protein precipitation method was used for the preparation of all the plasma samples (calibration standards and QC samples, and real plasma samples post-dose). Plasma sample aliquots (75 µL) were added with IS solution (50 µL, 200 ng/mL) and methanol (500 µL) in tubes, then vortex-mixed for 1 min, and centrifuged at 14,000× *g* for 10 min. The supernatant was transferred to another clean tube, and then evaporated to dryness under nitrogen at 40 °C. The dried residue was reconstituted in 100 µL of methanol-H_2_O (70:30, *v/v*) and centrifuged at 14,000× *g* for 5 min before analysis, and an aliquot of 5 µL was injected into LC-MS/MS systems for analysis.

### 3.7. Method Validation

The method was validated according to the USA Food and Drug Administration (FDA) bioanalytical method validation guidance [30].

#### 3.7.1. Specificity and Matrix Effect

Specificity was evaluated by analyzing the chromatograms of six different sources of blank rat plasma, as well as the corresponding spiked samples. Each blank sample was checked following the sample preparation procedures and under the chromatographic conditions described above to ensure no interference from plasma. Matrix effects were investigated in six different sources of blank rat plasma, and were calculated by comparing the peak area of post-extracted spiked samples with those of the standards containing equivalent amounts of PDC prepared in the mobile phase.

#### 3.7.2. Precision and Accuracy

Intra-day assay precision and accuracy were investigated by determining six replicates at four different QC levels on the same day. Inter-day assay precision and accuracy were investigated by determining six replicates at four different QC levels over three days. The acceptance criteria included accuracy within ±15% deviation (expressed by RE%) from the nominal values, except LLOQ, where it should be ±20% and a precision of ≤15% RSD, except for LLOQ, where it should be ≤20%.

#### 3.7.3. Linearity and Sensitivity

Calibration range covered the expected assay concentrations. Linear regression analysis was carried out on the calibration curve generated by plotting peak areas ratio of PDC/IS *versus* concentration of PDC. Six points of standard curves were constructed by least-squares linear regression analysis using a weighing factor of 1/x^2^. The LLOQ was defined as the lowest concentration of the analyte that can be determined with the RSD ≤20%.

#### 3.7.4. Recovery

The recovery of PDC and IS from rat plasma during extraction was investigated at four QC concentrations by comparing the peak area in rat plasma samples spiked with the analyte prior to extraction with those spiked post-extraction.

#### 3.7.5. Stability

Stability of PDC in plasma was tested by the analysis of six replicates of QC samples (*n* = 3) exposed to various storage conditions. To assess the short-term stability study, QC samples were kept at 25 °C for at least 4 h and samples were processed at different time points and were determined and compared with the freshly prepared QC samples. For the long-term stability, QC samples were determined after storage of the plasma samples at −20 °C for 2 months. For freeze-thaw stability, QC samples were subjected to three freeze/thaw cycles (room temperature to −20 °C). For post-preparation stability, the processed QC samples stored in the autosampler trials for 12 h at 4 °C, were analyzed and compared with the freshly prepared QC samples.

### 3.8. Pharmacokinetic Study

All experimental procedures and protocols were approved by the Animal Care and Use Committee of Jilin University (Changchun, China). All the rats were fasted for 12 h before the experiment, and were randomly divided into four groups (*n* = 6 per group). Group I and II were intragastrically administrated an oral dose of 42 and 84 mg/kg pure PDC, respectively. Group III and IV were intragastrically administrated an oral dose of 0.16 and 0.32 g/kg IRE, which was equivalent to 42 and 84 mg/kg PDC, respectively. Both pure PDC and IRE powder was suspended in 0.5% carboxymethyl cellulose sodium (CMC-Na) solution just before use. Blood samples (0.3 mL) were collected from the orbital sinus venous plexus at 0, 0.083, 0.25, 0.5, 0.75, 1, 1.5, 2, 3, 4, 6, 8, and 12 h after gavage dosing. After centrifuging at 5000× *g* for 10 min, the plasma samples were obtained and frozen at −20 °C until analysis.

### 3.9. Data Analysis

The pharmacokinetic parameters including *C*_max_, *T*_max_, *t_1/2z_*, *AUC_0–t_*, *AUC_0–∞_*, *CL*_z_/*F*, and *V*_z_/*F* were calculated by non-compartmental method using the DAS 2.0 pharmacokinetics program (Chinese Pharmacology Society, Beijing, China). An independent-sample *t*-test was performed twice to evaluate the differences of pharmacokinetic parameters between the two groups.

## 4. Conclusions

A rapid, selective, and sensitive LC-MS/MS assay for analyzing PDC in rat plasma was developed for the first time. Based on a survey of literature to date, there has been no report of the pharmacokinetic behavior of PDC, or a comparative pharmacokinetic study of PDC *in vivo* following the oral administration of pure PDC and IRE. The method was successfully applied to the pharmacokinetic study of the active component of *Ilex rotunda*, which will be helpful in providing a firm basis for the design of dosage regimens in pharmacodynamics experiments and preclinical safety evaluations.
